# *Hanseniaspora vineae* and the Concept of Friendly Yeasts to Increase Autochthonous Wine Flavor Diversity

**DOI:** 10.3389/fmicb.2021.702093

**Published:** 2021-08-04

**Authors:** Francisco Carrau, Paul A. Henschke

**Affiliations:** ^1^Área Enología y Biotecnología de Fermentaciones, Departamento Ciencia y Tecnología de Alimentos, Universidad de la Republica, Montevideo, Uruguay; ^2^The Australian Wine Research Institute, Adelaide, SA, Australia; ^3^School of Agriculture, Food and Wine, The University of Adelaide, Urrbrae, SA, Australia

**Keywords:** microbial terroir, mixed cultures, yeast consortia, low-input winemaking, minimal intervention

## Abstract

In this perspective, we will explain the concept of “friendly” yeasts for developing wine starters that do not suppress desirable native microbial flora at the initial steps of fermentation, as what usually happens with *Saccharomyces* strains. Some non-*Saccharomyces* strains might allow the development of yeast consortia with the native terroir microflora of grapes and its region. The positive contribution of non-*Saccharomyces* yeasts was underestimated for decades. Avoiding them as spoilage strains and off-flavor producers was the main objective in winemaking. It is understandable, as in our experience after more than 30 years of wine yeast selection, it was shown that no more than 10% of the isolated native strains were positive contributors of superior flavors. Some species that systematically gave desirable flavors during these screening processes were *Hanseniaspora vineae* and *Metschnikowia fructicola*. In contrast to the latter, *H. vineae* is an active fermentative species, and this fact helped to build an improved juice ecosystem, avoiding contaminations of aerobic bacteria and yeasts. Furthermore, this species has a complementary secondary metabolism with *S. cerevisiae*, increasing flavor complexity with benzenoid and phenylpropanoid synthetic pathways practically inexistent in conventional yeast starters. How does *H. vineae* share the fermentation niche with other yeast strains? It might be due to the friendly conditions it creates, such as ideal low temperatures and low nitrogen demand during fermentation, reduced synthesis of medium-chain fatty acids, and a rich acetylation capacity of aromatic higher alcohols, well-known inhibitors of many yeasts. We will discuss here how inoculation of *H. vineae* strains can give the winemaker an opportunity to develop ideal conditions for flavor expression of the microbial terroir without the risk of undesirable strains that can result from spontaneous yeast fermentations.

## Introduction

Traditionally, our ancestors produced wines by exploiting the indigenous yeast diversity present on grapes without knowledge of their winemaking capability. As wine quality was highly variable within and across different vintages, the challenge at that time was to obtain consistency and to search for vinification conditions that allowed greater standardization of their own wine styles. After Pasteur in 1866 showed that many yeast species rather than simple chemical reactions were responsible for wine fermentation (Barnett, 2000), wine masters from the beginning of the 20th century began to search for the “pure ferment” concept (Hansen, 1895; Regenberg and Hansen, 2001), so as to increase the reliability of vintage quality. This period saw the improvement of wine quality until the 1980s which promoted the successful growth of the wine industry globally and increased the consumption of better quality wine. Thanks to a considerable expansion of knowledge on the roles of yeast and bacteria in winemaking over the past 60 years (Ribereau-Gayon et al., 1951; Rankine, 1967; Amerine and Kunkee, 1968), wines became more flavorsome, especially more fruity, and less faulty than in the past (Lambrechts and Pretorius, 2000; Fleet, 2003; Ugliano and Henschke, 2009; Steensels and Verstrepen, 2014; Pretorius, 2020). However, in the 1990s, some winemakers and consumers noticed that wines in the general wine markets were becoming more uniform in flavor in terms of lacking complexity and diversity. In particular, the flavor characteristics of wines from major producing countries, which have specific terroirs, were becoming less apparent (Bisson et al., 2002). The majority of the wine producers were using the same conventional fermentation technology based on *Saccharomyces cerevisiae* (Jolly et al., 2014), which essentially precluded the opportunity for their respective microbial terroir to participate in the vinification process. This led some artisans to suggest that wine styles needed to return to their “grape roots” and develop the concept of microbial terroir flavors based on “low-input” winemaking strategies (Ramey, 1995). The challenge was to return to traditional winemaking technologies but guided by a greater wealth of knowledge compared with earlier times (Carrau, 2006; van Wyk et al., 2020). It is now well established that increased yeast diversity can contribute to the diversity of the volatile chemical composition of wine (Romani et al., 2020), which might increase the sensory diversity of wine flavor, a still controversial concept of sensory complexity (Varela et al., 2009; Smith, 2012; Köster and Mojet, 2016; Borren and Tian, 2021). Moreover, increased diversity can also results in process stability and productivity in microbial communities (Lehman and Tilman, 2000; Briones and Raskin, 2003). Whilst complex ecological studies are needed to prove this phenomenon in several fermentation niches, there is an increasing numbers of studies showing that mixed cultures can more effciently exhaust nutrients when compared with single cultures (Medina et al., 2012; Perez et al., 2020). This effect might further allow the reduction of microbial contamination during wine maturation. However, studies in real winemaking conditions at winery scale are very scarce (Romani et al., 2020), and when scaling up this technology of increased yeast diversity, the risks of appearance of undesirable flavors might result. At industrial sized fermentations, unpredictable interactions within a complex natural microflora, as the result of increasing reductive or oxidative conditions (Fariña et al., 2012; Varela et al., 2021) will demand careful daily process control by tasting. The attraction for food and wine is all about the flavor phenotype (Cordente et al., 2012; Carrau et al., 2015). These winemaking strategies are not romantic, and understanding deeply wine microbiology management is fundamental to obtain high-quality differentiated wines which reflect the region. Our proposal for developing friendly starters is based on careful strain selection by flavor and low nitrogen demand that was developed in order to obtain consistent screenings of superior native strains (Carrau et al., 2015). In our experience these aspects combined with an active but moderate fermentation capacity at temperatures below 20°C, resulted in wines of desirable flavors with certain yeast strains. By this selection strategy, we detected *Hanseniaspora vineae* at the initial steps of fermentation. In addition, we have characterized this yeast as having very different metabolic synthetic pathways compared with *Saccharomyces* that enrich wines with several grape flavor compounds related to the three aromatic amino acids such as benzenoids (Martin et al., 2016b; Valera et al., 2020a), other phenylpropanoids, and isoprenoids (Giorello et al., 2019; Del Fresno et al., 2020; Martin et al., 2021b). In this perspective, we will discuss the concept of “friendly” yeasts for developing wine starters and how the inoculation of *H. vineae* strains compared with conventional *Saccharomyces* strains gives the winemaker new tools to manage the microbial terroir flavor expression without the risk of unreliable spontaneous fermentations (Knight et al., 2020; Griggs et al., 2021).

## Friendly Yeasts Cooperate With the Microbial Terroir

**FIGURE 1:**
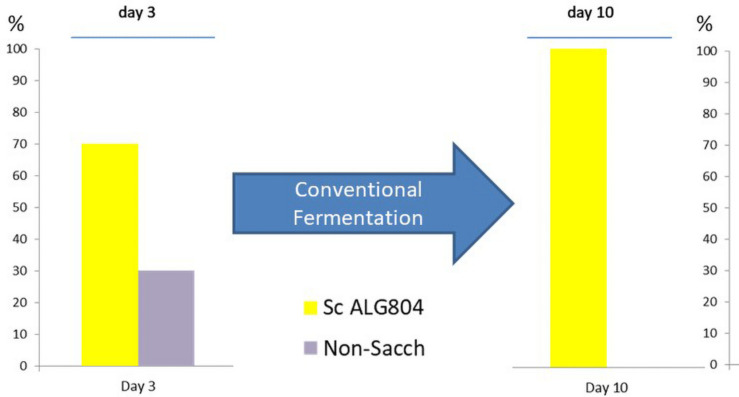
Under conventional wine production, winemakers initiate fermentation by inoculating with a selected commercial strain. Commercial inocula of *Saccharomyces* strains are well known for efficient initiation of fermentation and typically result in the exclusion of other strains during the course of fermentation [results shown were adapted from Medina et al. (2013)].

**FIGURE 2:**
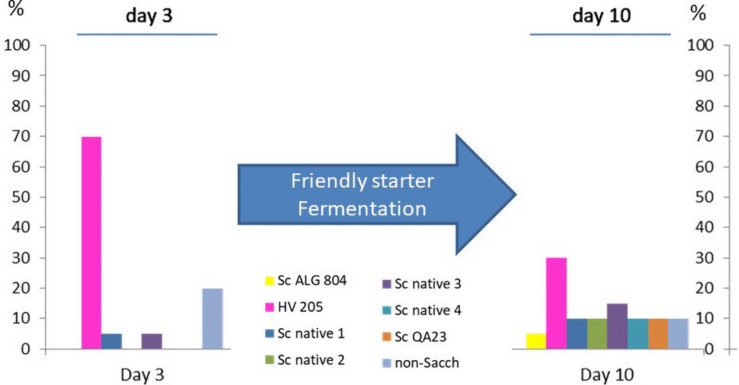
This shows an example of the yeast profile of a Chardonnay grape must inoculated with a single strain of HV025. This treatment shows an increase in yeast diversity during fermentation, as *H. vineae* allows the native or winery microflora to participate during fermentation. We apply the concept of “friendly starters” to a yeast inoculum that allows various yeast species and strains to share the fermentation environment.

It is well known that the majority of commercial yeasts added to a grape juice fermentation as starter culture rapidly control the process by reaching above 90% of the total yeast flora (Fleet, 1993; Medina et al., 2013; Carrau et al., 2020). This situation is common with *Saccharomyces* starters that are highly competitive compared with the native microflora, which we can defined as “selfish” or “unfriendly” strains (Rendueles and Ghigo, 2012). This species has evolved a highly competitive strategy for removing key nutrients in a grape juice, such as amino acids and vitamins, within a few hours (Alonso-del-Real et al., 2019). Furthermore, they actively use the glycolytic and alcohol fermentation pathways to exclude the native microflora of grape must niches, not only by producing ethanol and CO_2_ but also increasing temperature and producing compounds such as short- and medium-chain fatty acids, isoacids, or higher alcohols that can inhibit other yeast species (Goddard, 2008; Valera et al., 2019). These aroma compounds can be defined as “toxic” intercellular communication mechanisms, in contrast to the reduced production of these flavors by a friendly yeast strain. During the last decade or more, many non-*Saccharomyces* starters in mixed culture fermentation with a strain of *Saccharomyces* have been studied, usually inoculated in sequential mode, so as to enhance the opportunity of the non-*Saccharomyces* to influence the winemaking process compared with co-inoculations where its impact can be limited by exclusion or competition with *Saccharomyces* (Padilla et al., 2016; Aranda, 2019; Borren and Tian, 2021). In summary, the competitive advantage of *Saccharomyces* over the majority of non-*Saccharomyces* yeasts can reside in various stress mechanisms, including nitrogen depletion, sugar transporter adaptations to high osmotic pressure of grape juice (high sugar content) and to a more active proton-pump ATPase adapted to low pH and high ethanol (Ganucci et al., 2018; Palmgren and Morsomme, 2019). These key adaptations that are strain dependent can explain why *Saccharomyces* species and their related hybrids typically dominate the fermentation niche. Interestingly, in reference to secondary fermentation compounds, that are inhibitors of cell activity, we have noted that some characteristics of *H. vineae* metabolism can explain the resulting friendlier environment from its activity. Metabolic reactions such as its extreme capacity for higher alcohol acetylation represents a well-known mechanisms for detoxifing acetates and their corresponding alcohol in the fermentation medium (Peddie, 1990). Furthermore, we have determined that *H. vineae* produces significantly lower concentrations of fatty acids and has a slower rate of ethanol and CO_2_ production compared with *Saccharomyces* strains (Valera et al., 2020b). These attributes are characteristic of *H. vineae* within the apiculate group of the *Hanseniaspora* genus (Martin et al., 2018; Giorello et al., 2019; Valera et al., 2020a,b). During Chardonnay winemaking with *H. vineae* strains (Medina et al., 2013), we noted that in control-inoculated wines with *S. cerevisiae* ALG804, the native grape microflora was invariably dominated by this strain within several days. In Figure 1, we show a yeast profile of conventional wine fermentation inoculated with 10 g/Hl of active dry yeast, which clearly reveals numerical dominance by this species. In contrast, Figure 2 shows that inoculation with *H. vineae* HV025 permitted the participation of eight different strains at day 10 of the fermentation process. Although we can see in Figure 2 that two commercial strains appeared that might come from previous winemaking vintages, it is interesting to note that there were also four native *Saccharomyces* strains according to our yeast commercial data bank by DNA microsatellite profiles (Jubany et al., 2008).

## The Impact of Non-*Saccharomyces* Strains on Sensory Wine Differentiation

Few publications have focused on demonstrating increased sensory diversity associated with fermentation microflora using high-quality sensory techniques. Over the past two decades, many investigations have shown a great diversity of chemical compound analysis by GCMS of wines made with non-*Saccharomyces* and mixed species cultures compared with *Saccharomyces* yeasts. A large range of volatile compounds can be detected, but their impact on the sensory properties of wine is often lacking (Spence and Wang, 2019). The challenge today is to correlate wine chemical data with formal sensory analysis using for example a trained panel of judges or simple methods at the winery (Lesschaeve and Noble, 2010). Wines made with different yeast strains can often be consistently differentiated by sensory profiling techniques or can be judged as being sensorially more complex than others (Padilla et al., 2016; Binati et al., 2019). Many reports showing detailed chemical analyses will need to be validated with sensory analysis as they are merely showing just the chemical diversity of yeast metabolism in different winemaking conditions. Many of the reports on experimental vinification were performed with either chemically defined or natural grape fermentation media with either undefined or an excess concentration of yeast assimilable nitrogen (Carrau et al., 2017; Perli et al., 2020). Although it is clear that many of the recent non-*Saccharomyces* vinification studies have demonstrated an impact on wine quality in different grape varieties or under mixed culture conditions, there is still very limited information on the behavior of non-*Saccharomyces* strains at winery scale (Jolly et al., 2014; Comitini et al., 2017; Morata et al., 2020). Since 2007, white wines have been produced with *H. vineae* on a commercial scale (Medina et al., 2007) and could be sensorially differentiated from wines produced by conventional *Saccharomyces* fermentations (Medina et al., 2013; Lleixa et al., 2016; Martin et al., 2018; Del Fresno et al., 2020, 2021). However, although red wines made under these treatments can be differentiated by chemical techniques, the sensory differentiation or quality conclusions of these processes in red wine vinification at winery scale were less clear. Our observation is that young red wines might be differentiated more easily than strong body red wines and even less following barrel maturation. Chemically, red wines made with *H. vineae* showed the presence of increased concentrations of benzenoids and acetate esters when compared with conventionally vinified red wines. In the meantime, metabolic footprinting techniques of these wines (Howell et al., 2006) allowed us to show that *H. vineae* had contributed to the aromatic chemical composition of wine on an industrial wine fermentation scale (Martín et al., 2021a).

## Increased Yeast Diversity Increases Flavor Complexity

It is widely known that protection of biodiversity should be a main biological focus for conserving agricultural ecosystems. As we previously mentioned, it was demonstrated that increased biodiversity in a given ecosystem niche increased community stability and productivity (Lehman and Tilman, 2000). The flavor complexity concept is still not clear and some authors believe it is more of an increase of flavor compound diversity from a chemical point of view. However, the relevant concept should include the sensory complexity when we talk about fermented beverages (Tempère et al., 2018). Increased flavor diversity has been demonstrated by the use of mixed culture inocula by which increased flavor complexity from a sensory point of view could be achieved, for example, in Chardonnay (Soden et al., 2000; Medina et al., 2013) or Sauvignon Blanc wines (Anfang et al., 2009; Knight et al., 2018). More recently, sensory studies of mixed culture fermentations in some other white and red varieties, have been reported (Varela, 2016; Padilla et al., 2017; Hranilovic et al., 2018; Benito et al., 2019; Castrillo et al., 2019; Binati et al., 2020; Del Fresno et al., 2020; Romani et al., 2020; Muñoz-Redondo et al., 2021). In our experience, a chemically defined grape must medium with low assimilable nitrogen has allowed the selection of non-*Saccharomyces* and *Saccharomyces* strains with a combination of both low nitrogen demand and intense desirable flavors production (Carrau et al., 2015). This procedure contributed to the selection of a new generation of native yeast strains adapted for low-input winemaking strategies, where the addition of DAP can be avoided. It is known that ammonium salts inhibit the synthesis of some of the main aroma compounds of particular interest for the varietal character of some grapes, such as phenylpropanoids or sulfur thiols (Martin et al., 2016a). This strategy is expected to identify yeasts that can share fermentation medium nutrients as they will have decreased nitrogen demand characteristics and will ensure the development of clean flavors. However, further studies regarding unpredictable yeast interactions at winery scale are being carried out to better understand the appearance of sluggish fermentations in some rich sugar white grape musts such as Petit Manseng and Chardonnay grapes which would yield ethanol concentrations exceeding above 13% alcohol by volume (Carrau et al., 2020).

## Yeast Diversity From Native Environments to the Winery

**FIGURE 3:**
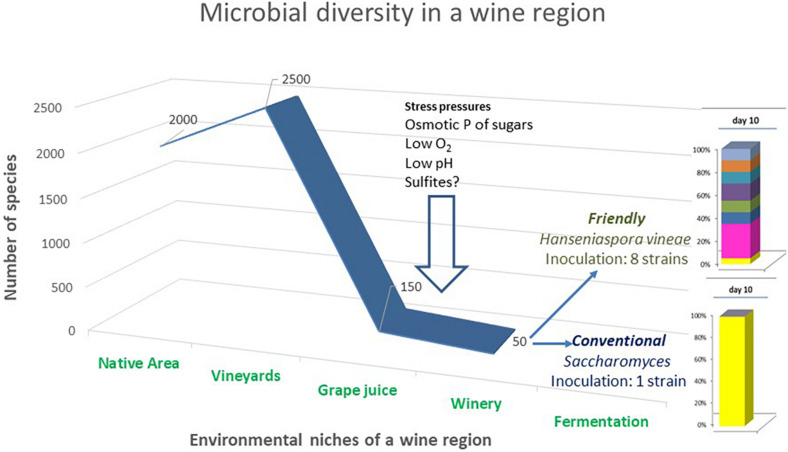
Yeast diversity along the different environments of a wine region ecosystem. Winemakers have the alternative to increase strain diversity when working with yeast strains that might share the fermentation niche with other native strains. The number of species data of the different niches was obtained from recent studies (Morrison-Whittle and Goddard, 2018), and fermentation data with *H. vineae* were adapted from Carrau et al. (2020).

The systematic use of *Saccharomyces* starter cultures in wine production has contributed to more uniform wine quality in the past 50 years. However, it is now considered by some that there is a limitation of flavor diversity due to this phenomenon affecting the development of new wine styles. This has led to a decrease in the flavor differentiation of wines from regional terroir sites which were previously described as having a “typical” flavor profile as associated with a given region (Liu et al., 2019, 2020). In Figure 3, we show the interesting process of how yeast diversity in a certain wine region is reduced from the vine to the final wine. This graph clearly shows that the addition of a pure culture inoculum of *Saccharomyces* at the initial step of fermentation in the winery restricts the rich microbial terroir flora to a single culture process.

Studies concerning yeast diversity in a wine region showed that the grape plants, including the fruit and soil niche ecosystems, lodge the higher number of yeast species compared with the native and winery environments (Morrison-Whittle and Goddard, 2018). Figure 3 shows the process from nature to the fermentation ecosystem revealing how yeast diversity undergoes a slight increase at the fruit ecosystem and, subsequently, a continuous decrease until the commencement of fermentation. Winemaker management of fermentation, that is interventions, can increase or even decrease yeast diversity to a single fermentation strain. In contrast to the rich biodiversity in a certain wine region, conventional inoculation of a pure *Saccharomyces* ferment finished with a single culture fermentation process.

## Discussion

The development of “friendly” yeasts can promote a sharing of the fermentation medium with some strains from natural environments so as to recover potential metabolic diversity along the process. The concept of introducing a starter with the characteristics described is based on the difficult task that would be incurred by a winery in selecting native yeasts with superior flavors from their vines, as it has been mentioned such strains might represent less than 10% of the total natural microflora. We have shown that a selection strategy for friendly yeast based on sensory analysis at low YAN concentrations and moderate fermentation capacity minimize the risk of sluggish processes or the production of undesirable flavors such as acetic acid, hydrogen sulfide, or acetaldehyde. *H. vineae* might also well fit the concept of friendly yeast owing to its low formation of higher alcohols and fatty acids compared with *Saccharomyces*, known inhibitors of many yeast strains. However, this approach to allowing the natural microflora to participate in the process should be controlled daily by tasting. However, in the wineries the fermentation increase microbial competition under such complex situations might be a small risk compared to the benefit of obtaining increased flavor complexity. The potential risks that could result from this approach are believed to be significantly smaller than the application of spontaneous fermentation processes. The future challenge for wine microbiologists and winemakers will be to understand fermentation from a holistic view point, so as to provide an alternative management of the process with the aim of minimizing the loss of strain diversity which would contribute to the loss of terroir characteristics. Strain interactions which increase strain diversity give infinite opportunities to explore flavor development. It is well known that in a complex consortia fermentation, there are many factors that can change the final flavor of a wine, such as size of the inocula, nutrient competition, metabolite–cell and cell–cell interactions, temperature, tank sizes, redox situations, and aeration. The development of friendly yeasts that share their environment with other strains is an interesting low-input winemaking strategy. This biotechnological approach might be considered a “romantic” way of producing particular or unique wines representing regionality. However, these strategies demand deep knowledge of microbiology and systematic tasting and sensory training at the winery to obtain quality and market differentiation.

## Data Availability Statement

The original contributions presented in the study are included in the article, further inquiries can be directed to the corresponding author.

## Author Contributions

FC wrote the article. PH wrote part of the article and reviewed the general presentation. Both authors contributed to the article and approved the submitted version.

## Conflict of Interest

The authors declare that the research was conducted in the absence of any commercial or financial relationships that could be construed as a potential conflict of interest.

## Publisher’s Note

All claims expressed in this article are solely those of the authors and do not necessarily represent those of their affiliated organizations, or those of the publisher, the editors and the reviewers. Any product that may be evaluated in this article, or claim that may be made by its manufacturer, is not guaranteed or endorsed by the publisher.

## References

[B1] Alonso-del-Real J. Pérez-Torrado R. Querol A. Barrio E. (2019). Dominance of wine *Saccharomyces cerevisiae* strains over *S. kudriavzevii* in industrial fermentation competitions is related to an acceleration of nutrient uptake and utilization. *Environ. Microbiol.* 21 1627–1644. 10.1111/1462-2920.14536 30672093

[B2] Amerine M. Kunkee R. (1968). Microbiology of winemaking. *Annu. Rev. Microbiol.* 22 323–358. 4879519 10.1146/annurev.mi.22.100168.001543

[B3] Anfang N. Brajkovich M. Goddard M. R. (2009). Co-fermentation with *Pichia kluyveri* increases varietal thiol concentrations in Sauvignon Blanc. *Aust. J. Grape Wine Res.* 15 1–8. 10.1111/j.1755-0238.2008.00031.x

[B4] Aranda A. (2019). Enological repercussions of Non-*Saccharomyces* Species. *Fermentation* 5:68. 10.3390/fermentation5030068

[B5] Barnett J. A. (2000). A history of research on yeasts 2: Louis Pasteur and his contemporaries, 1850–1880. *Yeast* 16 755–771. 10.1002/1097-0061(20000615)16:8<755::aid-yea587>3.0.co;2-4 10861901

[B6] Benito S. Ruiz J. Belda I. Kiene F. Beisert B. Navascués E. (2019). “Application of non-*Saccharomyces* yeasts in wine production,” in *Non-Conventional Yeasts: From Basic Research to Application*, ed. Sibirny A. (Cham: Springer), 75–89. 10.1007/978-3-030-21110-3_3

[B7] Binati R. L. Innocente G. Gatto V. Celebrin A. Polo M. Felis G. E. (2019). Exploring the diversity of a collection of native non-Saccharomyces yeasts to develop co-starter cultures for winemaking. *Food Res. Int.* 122 432–442. 10.1016/j.foodres.2019.04.043 31229097

[B8] Binati R. L. Junior W. J. L. Luzzini G. Slaghenaufi D. Ugliano M. Torriani S. (2020). Contribution of non-*Saccharomyces* yeasts to wine volatile and sensory diversity: a study on *Lachancea thermotolerans*, *Metschnikowia spp*. and *Starmerella bacillaris* strains isolated in Italy. *Int. J. Food Microbiol.* 318:108470. 10.1016/j.ijfoodmicro.2019.108470 31841784

[B9] Bisson L. F. Waterhouse A. L. Ebeler S. E. Walker M. A. Lapsley J. T. (2002). The present and future of the international wine industry. *Nature* 418 696–699. 10.1038/nature01018 12167877

[B10] Borren E. Tian B. (2021). The important contribution of non-*Saccharomyces* yeasts to the aroma complexity of wine: a review. *Foods* 10:13. 10.3390/foods10010013 33374550 PMC7822458

[B11] Briones A. Raskin L. (2003). Diversity and dynamics of microbial communities in engineered environments and their implications for process stability. *Curr. Opin. Biotechnol.* 14 270–276. 10.1016/S0958-1669(03)00065-X 12849779

[B12] Carrau F. (2006). “Native yeasts for low input winemaking: searching for wine diversity and increased complexity,” in *Proceedings of the International Wine Microbiology Symposium*, CSU International Wine Microbiology Symposium, (Tenaya Lodge, CA: California State University), 33–39.

[B13] Carrau F. Boido E. Dellacassa E. (2017). “Yeast diversity and flavor compounds,” in *Fungal Metabolites*, eds Mérillon J. M. Ramawat K. (Cham: Springer), 569–597. 10.1007/978-3-319-19456-1_32-1

[B14] Carrau F. Boido E. Ramey D. (2020). Yeasts for low input winemaking: microbial terroir and flavor differentiation. *Adv. Appl. Microbiol.* 111 89–121. 10.1016/bs.aambs.2020.02.001 32446413

[B15] Carrau F. Gaggero C. Aguilar P. S. (2015). Yeast diversity and native vigor for flavor phenotypes. *Trends Biotechnol.* 33 148–154. 10.1016/j.tibtech.2014.12.009 25630239

[B16] Castrillo D. Rabuñal E. Neira N. Blanco P. (2019). Oenological potential of non-*Saccharomyces* yeasts to mitigate effects of climate change in winemaking: impact on aroma and sensory profiles of Treixadura wines. *FEMS Yeast Res.* 19:foz065. 31584676 10.1093/femsyr/foz065

[B17] Comitini F. Capece A. Ciani M. Romano P. (2017). New insights on the use of wine yeasts. *Curr. Opin. Food Sci.* 13 44–49. 10.1016/j.cofs.2017.02.005

[B18] Cordente A. G. Curtin C. D. Varela C. Pretorius I. S. (2012). Flavour-active wine yeasts. *Appl. Microbiol. Biotechnol.* 96 601–618. 10.1007/s00253-012-4370-z 22940803 PMC3466427

[B19] Del Fresno J. M. Escott C. Loira I. Carrau F. Cuerda R. Schneider R. (2021). The impact of *Hanseniaspora vineae* fermentation and ageing on lees on the terpenic aromatic profile of white wines of the Albillo variety. *Int. J. Mol. Sci.* 22:2195. 10.3390/ijms22042195 33672220 PMC7926379

[B20] Del Fresno J. M. Escott C. Loira I. Herbert-Pucheta J. E. Schneider R. Carrau F. (2020). Impact of *Hanseniaspora vineae* in alcoholic fermentation and ageing on lees of high-quality white wine. *Fermentation* 6:66. 10.3390/FERMENTATION6030066

[B21] Fariña L. Medina K. Urruty M. Boido E. Dellacassa E. Carrau F. (2012). Redox effect on volatile compound formation in wine during fermentation by *Saccharomyces cerevisiae*. *Food Chem.* 134 933–939. 10.1016/j.foodchem.2012.02.209 23107710

[B22] Fleet G. H. (1993). “Yeast growth during fermentation,” in *Wine Microbiology and Biotechnology*, ed. Fleet G. H. (Chur: Harwood Academic Publishers), 27–54.

[B23] Fleet G. H. (2003). Yeast interactions and wine flavour. *Int. J. Food Microbiol.* 86 11–22. 10.1016/S0168-1605(03)00245-9 12892919

[B24] Ganucci D. Guerrini S. Mangani S. Vincenzini M. Granchi L. (2018). Quantifying the effects of ethanol and temperature on the fitness advantage of predominant *Saccharomyces cerevisiae* strains occurring in spontaneous wine fermentations. *Front. Microbiol.* 9:1563. 10.3389/fmicb.2018.01563 30057578 PMC6053494

[B25] Giorello F. Valera M. J. Martin V. Parada A. Salzman V. Camesasca L. (2019). Genomic and transcriptomic basis of Hanseniaspora vineae’s impact on flavor diversity and wine quality. *Appl. Environ. Microbiol.* 85:e01959–18. 10.1128/AEM.01959-18 30366992 PMC6293095

[B26] Goddard M. R. (2008). Quantifying the complexities of *Saccharomyces cerevisiae*’s ecosystem engineering via fermentation. *Ecology* 89 2077–2082. 10.1890/07-2060.1 18724717

[B27] Griggs R. G. Steenwerth K. L. Mills D. A. Cantu D. Bokulich N. A. (2021). Sources and assembly of microbial communities in vineyards as a functional component of winegrowing. *Front. Microbiol.* 12:836. 10.3389/fmicb.2021.673810 33927711 PMC8076609

[B28] Hansen E. C. (1895). Experimental studies on the variation of Yeast-cells. *Ann. Bot.* 9 549–560. 10.1093/oxfordjournals.aob.a090755

[B29] Howell K. S. Cozzolino D. Bartowsky E. J. Fleet G. H. Henschke P. A. (2006). Metabolic profiling as a tool for revealing *Saccharomyces* interactions during wine fermentation. *FEMS Yeast Res.* 6 91–101. 10.1111/j.1567-1364.2005.00010.x 16423074

[B30] Hranilovic A. Li S. Boss P. K. Bindon K. Ristic R. Grbin P. R. (2018). Chemical and sensory profiling of Shiraz wines co-fermented with commercial non-*Saccharomyces* inocula. *Aust. J. Grape Wine Res.* 24 166–180. 10.1111/ajgw.12320

[B31] Jolly N. P. Varela C. Pretorius I. S. (2014). Not your ordinary yeast: non-*Saccharomyces* yeasts in wine production uncovered. *FEMS Yeast Res.* 14 215–237. 10.1111/1567-1364.12111 24164726

[B32] Jubany S. Tomasco I. Ponce de Le n I. Medina K. Carrau F. Arrambide N. (2008). Toward a global database for the molecular typing of Saccharomyces cerevisiae strains. *FEMS Yeast Res.* 8 472–484. 10.1111/j.1567-1364.2008.00361.x 18294198

[B33] Knight S. J. Karon O. Goddard M. R. (2020). Small scale fungal community differentiation in a vineyard system. *Food Microbiol.* 87:103358. 10.1016/j.fm.2019.103358 31948613

[B34] Knight S. J. Klaere S. Morrison-Whittle P. Goddard M. R. (2018). Fungal diversity during fermentation correlates with thiol concentration in wine. *Aust. J. Grape Wine Res.* 24 105–112. 10.1111/ajgw.12304

[B35] Köster E. P. Mojet J. (2016). “Familiarity, monotony, or variety: the role of flavor complexity in food intake,” in *Flavor: From Food to Behaviors, Wellbeing and Health*, eds Etiévant P. Guichard E. Salles C. Voilley A. (Sawston: Woodhead Publishing), 10.1016/B978-0-08-100295-7.00013-X

[B36] Lambrechts I. G. Pretorius I. S. (2000). Yeast and its importance to wine aroma-A review. *South Afr. J. Enol. Vitic.* 21 97–129.

[B37] Lehman C. L. Tilman D. (2000). Biodiversity, stability, and productivity in competitive communities. *Am. Nat.* 156 534–552. 10.1086/303402 29587515

[B38] Lesschaeve I. Noble A. C. (2010). “Sensory analysis of wine,” in *Managing Wine Quality Viticulture and Wine Quality, Woodhead Publishing Series in Food Science, Technology and Nutrition*, ed. Reynolds A. G. (Amsterdam: Elsevier), 189–217. 10.1533/9781845699284.2.189

[B39] Liu D. Chen Q. Zhang P. Chen D. Howell K. S. (2020). The fungal microbiome is an important component of vineyard ecosystems and correlates with regional distinctiveness of wine. *Msphere* 5 e00534–20. 32817452 10.1128/mSphere.00534-20 PMC7426168

[B40] Liu D. Zhang P. Chen D. Howell K. S. (2019). From the vineyard to the winery: how microbial ecology drives regional distinctiveness of wine. *Front. Microbiol.* 10:2679. 10.3389/fmicb.2019.02679 31824462 PMC6880775

[B41] Lleixa J. Martin V. Portillo C. Carrau F. Beltran G. Mas A. (2016). Comparison of the performances of *Hanseniaspora vineae* and *Saccharomyces cerevisiae* during winemaking. *Front. Microbiol.* 7:338. 10.3389/fmicb.2016.00338 27014252 PMC4792884

[B42] Martín V. Boido E. Dellacassa E. Schneider R. Román T. Morata A. (2021a). “Application of *Hanseniaspora vineae* Hv025 in white and red grapes vinification on a winery scale,” in *Proceedings of the National Conference American.Society. Enology and Viticulture. June 23th*, Monterrey, CA.

[B43] Martin V. Boido E. Giorello F. Mas A. Dellacassa E. Carrau F. (2016a). Effect of yeast assimilable nitrogen on the synthesis of phenolic aroma compounds by *Hanseniaspora vineae* strains. *Yeast* 33 323–328. 10.1002/yea.3159 26945700

[B44] Martin V. Giorello F. Fariña L. Minteguiaga M. Salzman V. Boido E. (2016b). De Novo synthesis of Benzenoid compounds by the yeast *Hanseniaspora vineae* increases the flavor diversity of wines. *J. Agric. Food Chem.* 64 4574–4583. 10.1021/acs.jafc.5b05442 27193819

[B45] Martin V. Jose Valera M. Medina K. Boido E. Carrau F. (2018). Oenological impact of the *Hanseniaspora/Kloeckera* yeast genus on wines — A review. *Fermentation* 4:76. 10.3390/fermentation4030076

[B46] Martin V. Valera M. J. Medina K. Schneider R. Boido E. Carrau F. (2021b). “Application of Hanseniaspora vineae to improve white wine quality,” in *White Wine Technology*, Chap 9, 1st Edn, ed. Morata A. (Academic Press), 340.

[B47] Medina K. Boido E. Dellacassa E. Carrau F. (2012). Growth of non-*Saccharomyces* yeasts affects nutrient availability for *Saccharomyces cerevisiae* during wine fermentation. *Int. J. Food Microbiol.* 157 245–250. 10.1016/j.ijfoodmicro.2012.05.012 22687186

[B48] Medina K. Boido E. Fariña L. Gioia O. Gomez M. E. Barquet M. (2013). Increased flavour diversity of Chardonnay wines by spontaneous fermentation and co-fermentation with *Hanseniaspora vineae*. *Food Chem.* 141 2513–2521. 10.1016/j.foodchem.2013.04.056 23870989

[B49] Medina K. Ferreri L. Fariña L. Boido E. Dellacassa E. Gaggero C. (2007). Aplicación de la levadura *Hanseniaspora vineae* en cultivos mixtos con *Saccharomyces cerevisiae* en la vinificación. *Rev. Enol.* 4 1–6.

[B50] Morata A. Escott C. Bañuelos M. A. Loira I. Del Fresno J. M. González C. (2020). Contribution of non-*Saccharomyces* yeasts to wine freshness. A review. *Biomolecules* 10:34. 10.3390/biom10010034 31881724 PMC7022396

[B51] Morrison-Whittle P. Goddard M. R. (2018). From vineyard to winery: a source map of microbial diversity driving wine fermentation. *Environ. Microbiol.* 20 75–84. 10.1111/1462-2920.13960 29052965

[B52] Muñoz-Redondo J. M. Puertas B. Cantos-Villar E. Jiménez-Hierro M. J. Carbú M. Garrido C. (2021). Impact of sequential inoculation with the Non-*Saccharomyces T. delbrueckii* and *M. pulcherrima* combined with *Saccharomyces cerevisiae* strains on chemicals and sensory profile of Roseì wines. *J. Agric. Food Chem.* 69 1598–1609. 10.1021/acs.jafc.0c06970 33507745

[B53] Padilla B. Gil J. V. Manzanares P. (2016). Past and future of non-*Saccharomyces* yeasts: from spoilage microorganisms to biotechnological tools for improving wine aroma complexity. *Front. Microbiol.* 7:411. 10.3389/fmicb.2016.00411 27065975 PMC4814449

[B54] Padilla B. Zulian L. Ferreres À Pastor R. Esteve-Zarzoso B. Beltran G. (2017). Sequential inoculation of native non-*Saccharomyces* and *Saccharomyces cerevisiae* strains for wine making. *Front. Microbiol.* 8:1293. 10.3389/fmicb.2017.01293 28769887 PMC5513938

[B55] Palmgren M. Morsomme P. (2019). The plasma membrane H+-ATPase, a simple polypeptide with a long history. *Yeast* 36 201–210. 10.1002/yea.3365 30447028 PMC6590192

[B56] Peddie H. A. B. (1990). Ester formation in brewery fermentations. *J. Insitute Brew.* 96 327–331. 10.1002/j.2050-0416.1990.tb01039.x

[B57] Perez G. Debernardis F. Boido E. Carrau F. (2020). Simultaneous identification to monitor consortia strain dynamics of four biofuel yeast species during fermentation. *J. Ind. Microbiol. Biotechnol.* 47 1133–1140. 10.1007/s10295-020-02310-7 32965544

[B58] Perli T. Wronska A. K. Ortiz-Merino R. A. Pronk J. T. Daran J. M. (2020). Vitamin requirements and biosynthesis in *Saccharomyces cerevisiae*. *Yeast* 37 283–304. 31972058 10.1002/yea.3461 PMC7187267

[B59] Pretorius I. S. (2020). Tasting the terroir of wine yeast innovation. *FEMS Yeast Res.* 20:foz084. 31830254 10.1093/femsyr/foz084 PMC6964221

[B60] Ramey D. (1995). “Low input winemaking- let nature do the work,” in *Proceedings of the Australian Wine Industry Technical Conference*, eds Sas A. N. Stockley C. S. Johnstone R. S. Lee T. H. (Adelaide, SA: Winetitles), 26–29.

[B61] Rankine B. C. (1967). Influence of yeast strain and pH on pyruvic acid content of wines. *J. Sci. Food Agric.* 18 41–44. 10.1002/jsfa.2740180201

[B62] Regenberg B. Hansen J. (2001). A history of research on yeasts 3: Emil Fischer Eduard Buchner and their contemporaries, 1880-1900. *Yeast* 18 363–388. 10.1002/1097-0061(20010315)18:4<363::aid-yea677>3.0.co;2-r 11223946

[B63] Rendueles O. Ghigo J.-M. (2012). Multi-species biofilms: how to avoid unfriendly neighbors. *FEMS Microbiol. Rev.* 36 972–989. 10.1111/j.1574-6976.2012.00328.x 22273363

[B64] Ribereau-Gayon J. Peynaud E. Lafourcade S. (1951). Sur L’influence de L’aeration au cours de la fermentation. *Ind. Agric. Aliment.* 68 141–150.

[B65] Romani C. Lencioni L. Biondi Bartolini A. Ciani M. Mannazzu I. Domizio P. (2020). Pilot scale fermentations of *Sangiovese*: an overview on the impact of *Saccharomyces* and Non-*Saccharomyces* Wine Yeasts. *Fermentation* 6:63. 10.3390/fermentation6030063

[B66] Smith B. (2012). Perspective: complexities of flavour. *Nature* 486:S6. 10.1038/486S6a 22717402

[B67] Soden A. Francis I. L. Oakey H. Henschke P. A. (2000). Effects of co-fermentation with *Candida stellata* and *Saccharomyces cerevisiae* on the aroma and composition of Chardonnay wine. *Aust. J. Grape Wine Res.* 6 21–30. 10.1111/j.1755-0238.2000.tb00158.x

[B68] Spence C. Wang Q. J. (2019). Wine expertise: perceptual learning in the chemical senses. *Curr. Opin. Food Sci.* 27 49–56. 10.1016/j.cofs.2019.05.003

[B69] Steensels J. Verstrepen K. J. (2014). Taming wild yeast: potential of conventional and nonconventional yeasts in industrial fermentations. *Annu. Rev. Microbiol.* 68 61–80. 10.1146/annurev-micro-091213-113025 24773331

[B70] Tempère S. Marchal A. Barbe J. C. Bely M. Masneuf-Pomarede I. Marullo P. (2018). The complexity of wine: clarifying the role of microorganisms. *Appl. Microbiol. Biotechnol.* 102 3995–4007. 10.1007/s00253-018-8914-8 29552694

[B71] Ugliano M. Henschke P. A. (2009). “Yeasts and wine flavour,” in *Wine Chemistry and Biochemistry*, eds Moreno-Arribas M. V. Polo M. C. (New York, NY: Springer), 313–392. 10.1007/978-0-387-74118-5_17

[B72] Valera M. J. Boido E. Dellacassa E. Carrau F. (2020b). Comparison of the glycolytic and alcoholic fermentation pathways of *Hanseniaspora vineae* with *Saccharomyces cerevisiae* wine yeasts. *Fermentation* 6:78. 10.3390/fermentation6030078

[B73] Valera M. J. Boido E. Ramos J. C. Manta E. Radi R. Dellacassa E. (2020a). The Mandelate pathway, an alternative to the phenylalanine ammonia lyase pathway for the synthesis of benzenoids in ascomycete yeasts. *Appl. Environ. Microbiol.* 86 e701–e720. 10.1128/AEM.00701-20 PMC7440790 32561586

[B74] Valera M. J. Morcillo-Parra M. Á Zagórska I. Mas A. Beltran G. Torija M. J. (2019). Effects of melatonin and tryptophol addition on fermentations carried out by *Saccharomyces cerevisiae* and non-*Saccharomyces* yeast species under different nitrogen conditions. *Int. J. Food Microbiol.* 289 174–181. 10.1016/j.ijfoodmicro.2018.09.013 30253310

[B75] van Wyk N. von Wallbrunn C. Swiegers J. H. Pretorius I. S. (2020). Biotechnology of wine yeasts. *Encycl. Mycol.* 2 428–446. 10.1016/B978-0-12-819990-9.00007-X

[B76] Varela C. (2016). The impact of non-*Saccharomyces* yeasts in the production of alcoholic beverages. *Appl. Microbiol. Biotechnol.* 100 9861–9874. 10.1007/s00253-016-7941-6 27787587

[B77] Varela C. Cuijvers K. Van Den Heuvel S. Rullo M. Solomon M. Borneman A. (2021). Effect of aeration on yeast community structure and volatile composition in uninoculated chardonnay wines. *Fermentation* 7:97. 10.3390/fermentation7020097

[B78] Varela C. Siebert T. Cozzolino D. Rose L. Maclean H. Henschke P. A. (2009). Discovering a chemical basis for differentiating wines made by fermentation with ‘wild’ indigenous inoculated yeasts: role of yeast volatile compounds. *Aust. J. Grape Wine Res.* 15:238. 10.1111/j.1755-0238.2009.00054.x

